# Neural network-derived perfusion maps: A model-free approach to computed tomography perfusion in patients with acute ischemic stroke

**DOI:** 10.3389/fninf.2023.852105

**Published:** 2023-03-09

**Authors:** Umberto A. Gava, Federico D’Agata, Enzo Tartaglione, Riccardo Renzulli, Marco Grangetto, Francesca Bertolino, Ambra Santonocito, Edwin Bennink, Giacomo Vaudano, Andrea Boghi, Mauro Bergui

**Affiliations:** ^1^Division of Neuroradiology, Molinette Hospital, Turin, Italy; ^2^Department of Neurosciences, University of Turin, Turin, Italy; ^3^Department of Computer Science, University of Turin, Turin, Italy; ^4^Department of Radiology, University Medical Center Utrecht, Utrecht, Netherlands; ^5^Image Sciences Institute, University Medical Center Utrecht, Utrecht, Netherlands; ^6^Division of Neuroradiology, San Giovanni Bosco Hospital, Turin, Italy

**Keywords:** machine learning, convolutional neural network (CNN), CT-perfusion imaging, perfusion maps, stroke

## Abstract

**Objective:**

In this study, we investigate whether a Convolutional Neural Network (CNN) can generate informative parametric maps from the pre-processed CT perfusion data in patients with acute ischemic stroke in a clinical setting.

**Methods:**

The CNN training was performed on a subset of 100 pre-processed perfusion CT dataset, while 15 samples were kept for testing. All the data used for the training/testing of the network and for generating ground truth (GT) maps, using a state-of-the-art deconvolution algorithm, were previously pre-processed using a pipeline for motion correction and filtering. Threefold cross validation had been used to estimate the performance of the model on unseen data, reporting Mean Squared Error (MSE). Maps accuracy had been checked through manual segmentation of infarct core and total hypo-perfused regions on both CNN-derived and GT maps. Concordance among segmented lesions was assessed using the Dice Similarity Coefficient (DSC). Correlation and agreement among different perfusion analysis methods were evaluated using mean absolute volume differences, Pearson correlation coefficients, Bland-Altman analysis, and coefficient of repeatability across lesion volumes.

**Results:**

The MSE was very low for two out of three maps, and low in the remaining map, showing good generalizability. Mean Dice scores from two different raters and the GT maps ranged from 0.80 to 0.87. Inter-rater concordance was high, and a strong correlation was found between lesion volumes of CNN maps and GT maps (0.99, 0.98, respectively).

**Conclusion:**

The agreement between our CNN-based perfusion maps and the state-of-the-art deconvolution-algorithm perfusion analysis maps, highlights the potential of machine learning methods applied to perfusion analysis. CNN approaches can reduce the volume of data required by deconvolution algorithms to estimate the ischemic core, and thus might allow the development of novel perfusion protocols with lower radiation dose deployed to the patient.

## 1. Introduction

Occlusion of a cerebral artery causes the sudden decrease of the blood perfusion in the vascular territory matching the occluded vessel. The peripheral regions of the area affected by the vascular occlusion have their blood flow deficit reduced by the collateral circulation, in comparison to the center of the affected territory. Ischemic lesions develop rapidly, originating from the center of the occluded vascular territory and progressively expanding to most peripheral regions.

From the onset of symptoms, in the ischemic hypo-perfused area of the brain two different regions may be identified: a central “core,” and a peripheral “penumbra,” where the former corresponds to the area of irreversible damage, and the latter to the area of a potential recovery, provided recanalization of the occluded vessel. Therefore, identification of core and penumbra may predict the fate of the tissue and drive reperfusion treatments (Donahue and Wintermark, 2015; Wannamaker et al., 2018). The extension of core and penumbra may be estimated using perfusion techniques, in particular CT Perfusion (CTP). During CTP, a series of low-dose scans is acquired after contrast bolus injection, allowing for computing time-density curves; deconvolution of these curves allows for generating parametric maps to track perfusion parameters dynamics. Cerebral Blood Volume (CBV), Cerebral Blood Flow (CBF), time to peak (TTP), time to peak of the deconvolved tissue residue function (Tmax), and mean transit time (MTT) are the estimated perfusion parameters most frequently used in clinical practice. Ischemic core and penumbra can be predicted by assessing perfusion parameters mismatch across different parametric maps, i.e., brain tissues characterized by reduced CBV and CBF, and increased MTT or TTP are interpreted as core lesions (Konstas et al., 2009a,b; Campbell et al., 2011).

Tmax has been found to approximate both penumbra and core identified with an increasing tissue perfusion delay (Demeestere et al., 2020).

The role of CTP is of particular importance in patients with unknown time from the onset of symptoms, or out of the 4.5- and 6-hours’ window used to select patients for intra-venous and intra-arterial reperfusion treatments, respectively. Two trials (DAWN and DEFUSE) demonstrated the clinical usefulness of intra-arterial reperfusion in patients selected using CBV and CBF estimation based on CTP (Albers et al., 2018; Nogueira et al., 2018).

Different algorithms are used to perform deconvolution of time-intensity curves, some of which are not public and may produce largely different maps (Kudo et al., 2010). In an ideal setting of limited noise, low variance, and no motion artifacts, a pixel-by-pixel analysis, as performed by deconvolution-based algorithms, is probably the best choice to obtain realistic, affordable, and reproducible maps. In a realistic setting, however, redundant information is acquired in order to overcome problems due to noise, large variance and movement. In practice, this imposes obtaining more slices, requiring a larger number of acquisitions and more X-ray exposure for the patients, to perform a series of spatial pre-processing steps for noise and variance reduction and to extract the arterial input function (AIF).

Luckily, Machine Learning (ML) approaches offer several potential advantages over canonical algorithms when applied to the problem of time-intensity curves deconvolution. In fact, ML techniques allow extracting information that is relatively insensitive to noise, misalignments, and intra-subjects variance (Dashtbani Moghari et al., 2021a; Li et al., 2021a).

Several supervised and unsupervised machine learning algorithms–e.g., Support Vector Machines, Random Forests, Ridge Regression, Feed forward Neural Network - have been applied to CTP and MRI perfusion showing better performance compared to simpler linear models (McKinley et al., 2018; Cheng et al., 2021).

In our study we explored whether a properly trained Convolutional Neural Network (CNN), based on a U-Net-like structure, can generate informative, AIF-independent, parametric maps of CBV, CBF, and time to peak TTP on a pre-processed dataset of CTP images. CTP images were obtained from a real-world dataset of patients with acute ischemic stroke (AIS), no large lesions on non-contrast CT scans, and candidates for reperfusion therapies (Barber et al., 2000). This dataset was chosen because it corresponds to the one used in the DAWN and DEFUSE trials, for which perfusion studies drive reperfusion therapies. We released publicly two versions of this dataset for data sharing following the FAIR protocol [UniToBrain, doi: 10.5281/zenodo.481760, doi: 10.21227/x8ea-vh1, (Gava et al., 2021)].

The datasets and the CNN training were developed as a use case of the European project DeepHealth^1^: a framework envisioned to tackle the real needs of the health sector and facilitate the daily work of medical personnel and expert users in terms of image processing and the use and training of predictive models without the need of combining numerous tools. To this end, the project will combine open High-Performance Computing infrastructure with ML techniques to support biomedical applications that require the analysis of large and complex biomedical datasets.

## 2. Materials and methods

All procedures performed in this study involved human participants and thus followed the ethical standards of our institutional review board (Comitato Etico Interaziendale, CEI, id number 596.345), and the 1964 Helsinki declaration and its later amendments or comparable ethical standards. The requirement for written informed consent was waived because of the retrospective nature of the study.

### 2.1. Clinical data

CT Perfusion data gathered for this study is part of a larger open-access dataset developed for the DeepHealth project which now includes 258 consecutive patients, obtained retrospectively from hospital Picture Archiving and Communication System (PACS) and is available for download (Gava et al., 2021).

Perfusion data from a subset of 115 patients were extracted. For training the CNN, 115 subjects were randomly split into a training set of 100, and a testing set of 15, used to compare our results to a gold standard method.

CT Perfusion acquisition parameters were as follows: Scanner GE 64 slice, 80 kV, 150 mAs, 44.5-s duration, 89 volumes (40 mm axial coverage) with slice thickness 5 mm, injection of 40 ml of Iodine contrast agent (300 mg/ml) at 4 ml/s speed.

### 2.2. Calculation of ground truth maps

We calculated perfusion maps, including CBF, CBV, and TTP, using a pipeline of spatial pre-processing and a state-of-the-art fast model-based non-linear regression (NLR) method developed by Bennink et al. (2016).

Motion correction was done using Elastix 5.0.1, by rigid registration of all CTP frames to the first frame (Bennink et al., 2016). Registration was initiated on a coarse resolution level (8× down-sampling), followed by a full-resolution level. The sum of squared differences between the frames was minimized using stochastic gradient descent with, respectively 2,000 and 8,000 samples and 300 iterations per resolution level.

After motion correction, the CTP frames were filtered using a 3D bilateral filter, guided by the mean of all CTP frames (Klein et al., 2010). The spatial kernel size (standard deviation) was 3 mm and the range kernel size 20 Hounsfield units.

The CBV, CBF, and TTP were estimated by fitting a model to the measured attenuation curve in each voxel by means of non-linear regression. The model convolves the measured AIF with a box-shaped impulse response function (IRF). The CBF determines the height of the box, the MTT determines its width and the TTP determines its position on the time-axis (Tomasi and Manduchi, 1998). A downhill-simplex algorithm was used to minimize the sum of squared errors in 300 iterations. The initial CBF, MTT, and TTP were 60 mL/100 g/min, 5 s, and 3.5 s, respectively.

Arterial input function and Venous output function (VOF) calculations were done automatically on a 100 voxels sample.

The box-shaped model developed by Bennink et al. (2016) describes the impulse response function (IRF) of the perfused tissue in terms of CBV, MTT, and tracer delay. The box-shaped IRF enables fast NLR analysis, which is critical in a clinical setting such as ischemic stroke.

The time attenuation curve of the tissue and the relative CBV, CBF, and TTP maps were estimated using the AIF in conjunction with the IRF.

### 2.3. CNN training

The filtered and registered images were the only input provided to the U-Net-like architecture. This network architecture has been originally developed for image segmentation, however, it proved to be robust and performed well in other scenarios as well (Chen et al., 2018; Falk et al., 2019). The original architecture was proposed by Ronneberger et al. (2015) and consisted of 18 convolutional layers with 3 × 3 filters, 4 up-convolution layers with 2 × 2 kernels, 4 max-pool layers with 2 × 2 kernels, and 1 convolutional layer with 1 × 1 convolution. Differently from similar models, U-Net is able to extract features at different spatial resolutions and, thanks to its “copy and crop” connections which resemble the residual connections in ResNets (He et al., 2016), the model automatically selects the optimal resolution(s) to extract features for the target training task, disregarding the other scales. In the original work, the authors proposed an extraction with five different resolutions (from 572 × 572 down to 32 × 32), which required a large number of filters per layer, from 64 at the highest resolution to 1,024 at the lowest.

To apply it to our problem, we needed to introduce modifications at the architectural level to fit our problem.

For segmentation tasks a state-of-the-art choice is to use max-pooling layers for sub-sampling:


y=max⁢(x⁢_⁢1,x⁢_⁢2,…,x⁢_⁢N)

**FIGURE 1:**
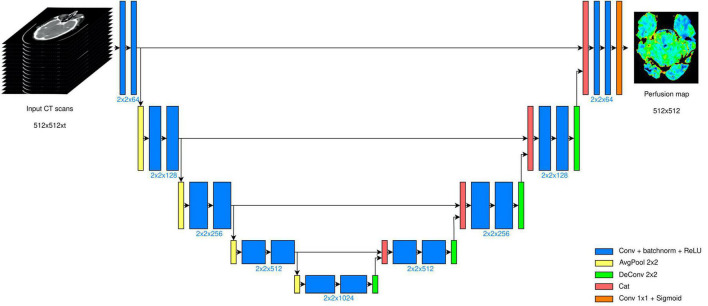
U-Net architecture deployed. The model takes scans of size 512 × 512, acquired in t different time instants. The bottleneck layer is placed after four encoding stages, and the output is a 512 × 512 map.

This operator, however, introduces a non-linear behavior that prevents the forward propagation of a great part of the information content (Sabour et al., 2017). Moreover, since the use of the standard max-pool layer in our context was suboptimal as we do not expect sparse features to be extracted, we employed average pooling layers in place of max-pool. By the same token, we used 2 × 2 kernels instead of 3 × 3 kernels not only for the average pooling and for the up-convolution, but also for the convolutional layers. The input to our model then were 512 × 512× t image stacks (in place of 572 × 572 × 3), where t defines the number of CT volumes acquired. The CT scans were processed, hence, as 3D tensors, where the third dimension is time. Depending on the chosen time granularity, the number of input channels changed accordingly. The overall CNN structure is displayed in Figure 1.

During training, instead of using standard cross-entropy loss, or dice score/focal loss, which are typical in segmentation tasks, we minimized the mean squared error loss (MSE), which was compatible with the desired ground truth output. No additional information (like the AIF) was provided to the CNN: all the information is implicitly extracted or inferred from the registered CT scans.

The model was pre-trained on 128 × 128 sub-sampled inputs for 250 epochs, after that it was fine-tuned for 50 additional epochs on the full 512 × 512 resolution. Training on full resolution images requires high consumption of GPU memory and the time needed to reach model convergence becomes very high.

Training on down-sampled images, on the contrary, allows us to reach a solution in a much shorter time since the task is simplified. The pre-training step produces a suitable initialization for the model: this type of approach is often used for training generative networks, and the results are comparable and smoother than those produced with very high dimensional image training. The training of the entire model for each target is done using Adam with a learning rate 10^–5^, b_1_ = 0.9, b_2_ = 0.999, and batch size equal to 8.

**FIGURE 2:**
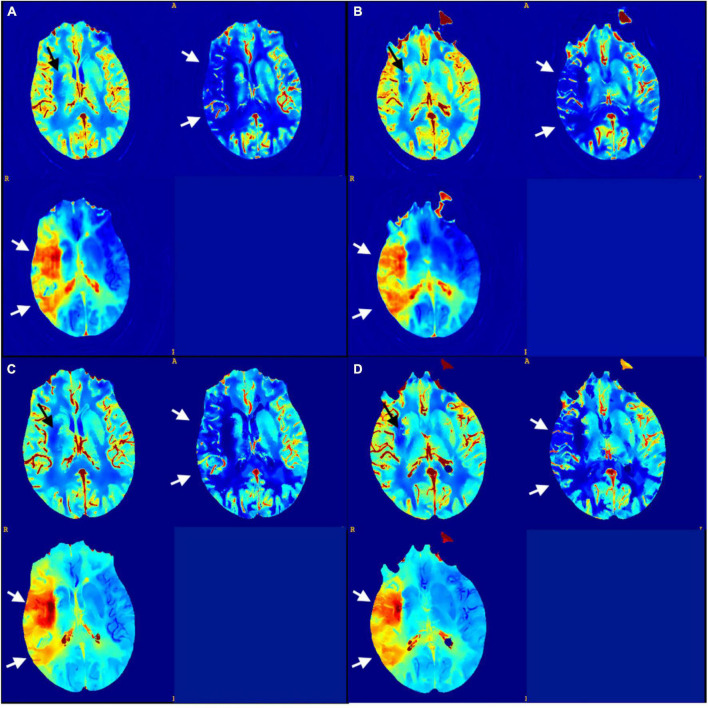
**(A,B)** CNN output maps from the testing set (CBV, CBF, and TTP); **(C,D)** matching sections of GT maps. There is a small infarct core displayed in the CBV map at the right basal ganglia (black arrows) and an extended penumbra showed in the CBF and TTP maps across right middle cerebral artery territories (white arrows).

The output of the model was a 512 × 512 map (Figure 2), where all the pixel values were normalized in the range 0–1. The entire model was trained using an SGD optimization strategy with a learning rate decay policy self-tuned according to the performance on the validation set. The source code is publicly available at https://github.com/EIDOSLAB/Neural-Network-derived-perfusion-maps.

To evaluate the performance of the CNN model in generating new maps, we implemented a threefold cross-validation protocol on the training dataset.

### 2.4. CNN performance

The accuracy of CNN maps was carried out through the segmentation of the infarct core (CBV) and total hypo-perfused territory (CBF, TTP), on the test set of 15 CNN-parametric maps. The process is performed manually by two expert radiologists using ITK-SNAP open-source software (Yushkevich et al., 2006). Segmentation was carried out section-wise following the axial direction.

To avoid bias induced by repetitive evaluation of the same patients, GT-maps segmentation was performed by two different radiologists.

**FIGURE 3:**
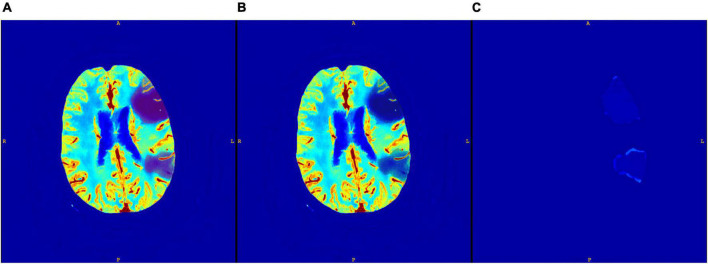
Core segmented on CNN CBV map **(A)**; core segmented on GT CBV map **(B)**; and the difference between the two maps **(C)**.

An example of a core segmentation on both CNN and GT maps is displayed in Figure 3. We notice that the map is perfectly reconstructed everywhere, except in the core region, where the CNN generated map has a positive, uniform bias, which however does not compromise the segmentation of the core area.

Penumbra volumes correspond to the mismatch between the total hypo-perfused region and the ischemic core.

The CNN segmented volumes from both raters were matched with the GT to assess overlapping regions by calculating the Dice Similarity Coefficient (DSC).

Dice Similarity Coefficient was also calculated by matching CNN segmentations from different raters of the same set of maps to evaluate inter-rater concordance.

Pearson correlation coefficient (r) was used to assess the relationship between the lesion volumes on GT and CNN maps. Bland-Altman and coefficient of repeatability (CR) analysis was also performed across volumes segmented on CNN and GT maps to assess agreement between the different methods.

Intraclass correlation coefficient (ICC) was used to assess reliability across volumes segmented from different raters.

Friedman test was used to look into significant differences across volumes of segmented lesions.

Statistical analysis was performed using the 3D-convert toolbox from ITK-SNAP, intraclass_corr function in Python 3.6.5 (ICC1k, pingouin library),^2^ and Statistical Package for the Social Sciences (SPSS) software (version 27.0.1.0).

## 3. Results

**FIGURE 4:**
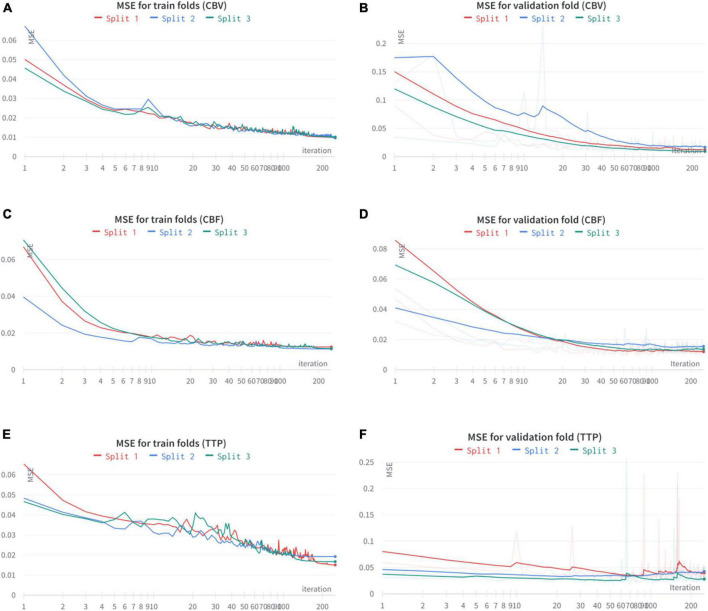
Train and validation curves for the threefold cross-validation results on CBV **(A,B)**, CBF **(C,D)**, and TTP **(E,F)**.

In Table 1 we reported the final performance of the models as both the MSE evaluated on the training set and the one obtained on the validation set. We would like to remark that the threefold slit was performed on the patient’s base. The MSE for both the generation of CBV and CBF is very low, in the order of 0.01 (corresponding to a PSNR close to 20 dB), having a generalization gap of 0.001 only. This shows that these two maps can be produced with very high fidelity, even on new subjects. Differently from the previous two maps, TTP’s generation results more problematic, with an average MSE of 0.017 and a higher generalization gap. Given the extremely high non-linearity of TTP maps, a worse generation performance is expected. All the training and validation results are shared in Figure 4.

**TABLE 1:** Threefold cross-validation results for CBV, CBF, and TTP.

Metric		MSE train folds	MSE validation fold
CBV	Split 1	0.010	0.010
	Split 2	0.009	0.014
	Split 3	0.010	0.009
	Average	0.010 ± 0.001	0.011 ± 0.003
CBF	Split 1	0.012	0.010
	Split 2	0.011	0.015
	Split 3	0.011	0.014
	Average	0.012 ± 0.001	0.013 ± 0.002
TTP	Split 1	0.015	0.044
	Split 2	0.019	0.037
	Split 3	0.017	0.024
	Average	0.017 ± 0.002	0.035 ± 0.010

Resulting MSE for CBV, CBF, and TTP CNN maps.

A total of 3 out of 15 CTP datasets used as CNN testing exhibited normal perfusion parameters on both GT and CNN-parametric maps and resulted in negative vessel occlusion on CT Angiography (CTA). Normal perfusion maps were excluded from DSC analysis to avoid overestimation of the segmentations comparison results.

**FIGURE 5:**
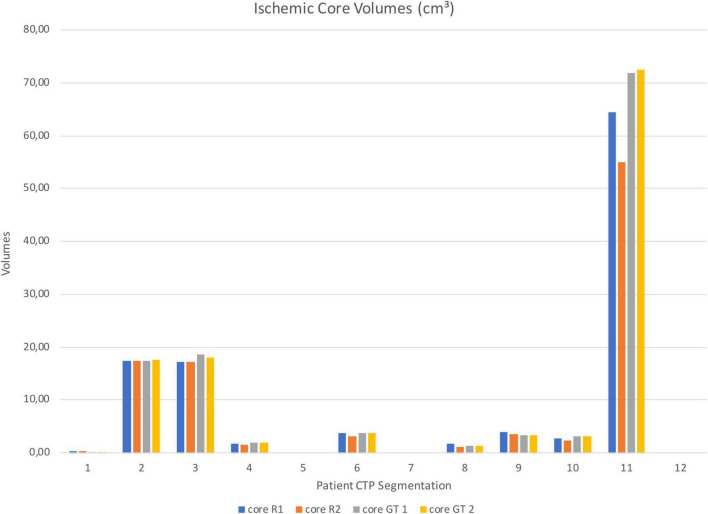
Test sample ischemic core segmentation volumes from Rater 1 (R1), Rater 2 (R2), GT 1 and GT 2.

**FIGURE 6:**
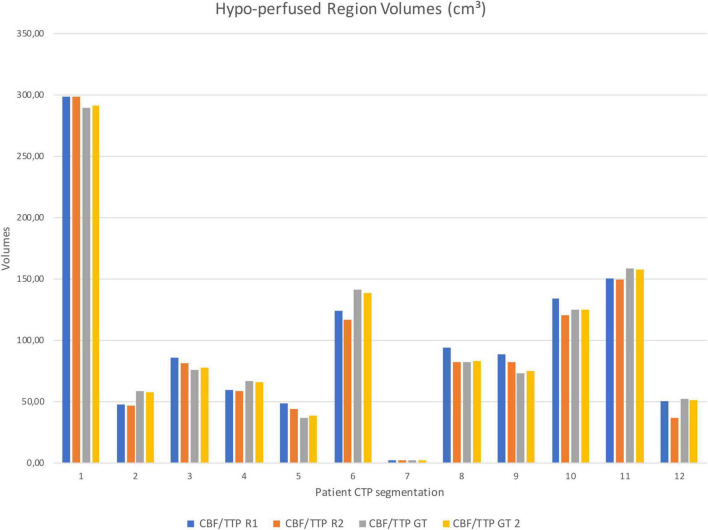
Test sample CBF/TTP segmentation volumes from Rater 1 (R1), Rater 2 (R2), GT 1 and GT 2.

Segmented core (CBV) and hypo-perfused regions (CBF/TTP) volumes of the remaining patients were shown in Figures 5, 6; 3 out of 12 patients presented with hypo-perfused territories without ischemic cores.

Mean DSC for all CBV lesions ranged from 0.80 to 0.86 and for CBF/TTP lesions from 0.82 to 0.87, with high DSC values are found both across and within different methods of perfusion analysis. DSC resulting from segmentation matching are presented as mean and standard deviation (SD) in Table 2.

**TABLE 2:** Average DSC and lesion volumes Pearson correlations.

DSC and Pearson correlation	CBV	CBF/TTP
Rater 1/GT 1 (DSC)	0.82 ± 0.07	0.85 ± 0.07
Rater 2/GT 1 (DSC)	0.79 ± 0.05	0.83 ± 0.09
Rater 1/GT 2 (DSC)	0.81 ± 0.07	0.84 ± 0.05
Rater 2/GT 2 (DSC)	0.80 ± 0.06	0.82 ± 0.07
Rater 1/Rater 2 (DSC)	0.85 ± 0.10	0.87 ± 0.06
GT1/GT 2 (DSC)	0.86 ± 0.05	0.85 ± 0.07
Rater 1/GT 1 Pearson *r*	0.99	0.98
Rater 2/GT 1 Pearson *r*	0.99	0.99
Rater 1/GT 2 Pearson *r*	0.98	0.99
Rater 2/GT 2 Pearson *r*	0.99	0.99

Resulting DSC of CBV and CBF/TTP segmentation expressed as mean and SD; Pearson correlation between CBV-CBF/TTP volumes on GT and CNN maps.

Friedman test did not reveal a significant difference among segmented volumes on CBV and CBF/TTP maps. Mean ICC for absolute agreement were excellent for volumes segmented both on CBV and CBF/TTP maps (0.98 C.I. 0.95–1.00; 0.99 C.I. 0.98–1.00).

We also found a strong positive correlation (*r* = 0.99, *r* = 0.98 with *p* < 0.001) between CBV–CBF/TPP lesion volume on GT and CNN maps for both raters (Table 2).

**FIGURE 7:**
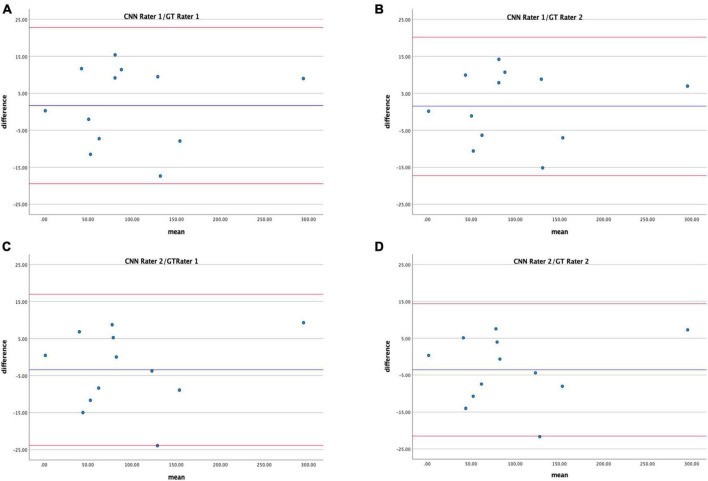
Bland–Altman plots showing agreement between total hypo-perfuse volume measurements derived from CNN and GT maps for Rater 1 **(A,B)** and Rater 2 **(C,D)**.

Bland–Altman analysis displays good agreement between the CNN proposed method and the GT in estimating hypo-perfused regions on CTP maps (Figure 7); CR for Rater 1 and Rater 2 were 19.9–17.7 and 20.9–18.4.

Mean absolute volume differences across the ischemic core from different observers and the GT were 1.20 ± 2.31 cm^3^ (Rater 1) and 2.27 ± 5.44 cm^3^ while mean volume differences for the whole hypo-perfused region were 9.37 ± 4.84 cm^3^ (Rater 1) and 8.52 ± 6.49 cm^3^ (Rater 2).

## 4. Discussion

The study demonstrated that parametric maps generated by our CNN-based approach are comparable to maps resulting from a state-of-the-art CTP NLR algorithm when working on pre-processed images.

There is a high DSC and strong linear correlation between CNN and GT segmented volumes.

The performance of the CNN at estimating ischemic core and penumbra is comparable to a state-of-the-art CTP NLR algorithm without additional inputs such as AIF or VOF.

### 4.1. Is AIF selection mandatory?

To precisely estimate perfusion parameters, the proposed CNN requires only registered CT scans, while deconvolution-based CT and MRI brain perfusion analysis methods need additional inputs, such as the AIF curve measured in a large feeding artery (Fieselmann et al., 2011). This suggests that our CNN is capable of combining information from arterial and tissue density to obtain quantitative estimates for the CBV, CBF, and MTT. On the other hand, other recent automated CTP analysis methods such as RAPID (Campbell et al., 2015), use AIF and the venous output function (VOF) from a major venous system to compute perfusion parameters and subsequently estimate ischemic core and penumbra (Laughlin et al., 2019).

In a model developed by Hess et al. (2018) the performance of the CNN in regressing the Tmax parameter on DSC-MRI perfusion imaging increases with the addition of bolus information. Future regression of the Tmax using our CNN could show if the assumption made by Hess et al. (2018) for MRI perfusion imaging also extend to CTP.

### 4.2. Translation to a general population

The CTP datasets were obtained from a population of patients eligible for reperfusion therapy, with limited or no hypodense lesions on non-contrast CT.

Such a population was targeted to simulate the clinical setting where CTP is a key parameter for clinical decisions: when the time of onset is not known, CTP allows to effectively select patients for treatment, as shown in DAWN and DEFUSE-3 trials (Albers et al., 2018; Nogueira et al., 2018). Our method shows a highly accurate performance on patients with no vessel occlusion on CTA and normal CTP parameters on GT maps, therefore suggesting that our CNN-based approach can yield highly reliable results even within the general population. Moreover, hypodense lesions may mark an ischemic core on CTP; this can introduce additional information to be exploited by our CNN-based approach without additionally estimating the tissue-curve offset, as required by deconvolution-based algorithms.

### 4.3. Limitations

The CNN was tested on a high dose/signal-to-noise ratio (SNR) dataset with limited axial coverage, thus an application to noisier datasets is required for a complete comparison with state-of-the-art techniques. Moreover, in order to confirm its robustness, our approach should undergo further testing on a larger population sample and use the latest CTP protocols from different scanners.

### 4.4. Applications and developments

Considering the highly similar performance of CNNs and deconvolution-based algorithms, one might ask why the former approach might be preferable. ML algorithms have been largely proven to overcome conventional image processing algorithms in practically every field [segmentation (Zhou et al., 2017; Clèrigues et al., 2019; Kloenne et al., 2020), noise reduction (Chen et al., 2017; Xiao et al., 2019; Kloenne et al., 2020; Dashtbani Moghari et al., 2021a), novelty detection (Chen et al., 2017; Zhang et al., 2019), radiation dose reduction (Chen et al., 2017; Dashtbani Moghari et al., 2021b; Li et al., 2021a)] and in recent years its use is expanding also to CTP and MRI perfusion imaging. In particular, the generation of synthetic maps using ML approaches has been performed with MRI DSC perfusion by Ho et al. (2016) and Meier et al. (2019). Meier et al. (2019) obtained results similar to ours: they compared the performance of a commercial FDA-approved perfusion software and a CNN not only to generate Tmax MRI perfusion maps, but also to identify selection criteria for reperfusion therapies. They concluded that CNN-based approaches may lead to greater standardization, a faster analysis pipeline, and increased robustness (Meier et al., 2019). Ho et al. (2016), instead, estimated voxel-wise MRI perfusion parameters using a deep learning approach exploiting the concentration time curve and AIF as inputs. Their approach, however, proved to be time-consuming and thus not ideal for clinical practice.

de la Rosa et al. (2021) developed a novel supervised CNN designed for estimating vascular function (AIF and VOF) in perfusion imaging, which showed improved CTP results when combined with traditional deconvolution algorithms. Their CNN used CTP 4D CTP data as inputs to generate the AIF and VOF curve associated with a probability map showing the voxel-wise contribution to the estimated parameters (de la Rosa et al., 2021).

In the ISLES-2018, challenges Song was ranked first using a deep learning method based on a U-Net architecture to produce synthetic diffusion-weighted imaging (DWI) images by combining information from CTP raw data and post-processed parametric maps. The CNN-derived DWI images were compared to the DWI GT using a discriminator to determine whether the presented image was synthetic or not. The information useful to determine the origin of the DWI maps were fed back into the deep-learning algorithm to improve the prediction of the synthetic DWI images (Hakim et al., 2021).

In a recent study Kuang et al. (2020) developed a ML model based on a random forest classifier to predict ischemic tissue probability in each voxel. They combined different CTP parameters with specific clinical time variables (onset to imaging and imaging to reperfusion). Their threshold-free model proved to perform better than the current clinically used method with fixed thresholding (Kuang et al., 2020). Similarly, Robben et al. (2019) showed that a deep learning approach using in combination native CTP images with metadata such as time parameters and treatment outcome was able to effectively predict the final infarct probability.

In their work, Dolz et al. (2018) propose a “multipath dense U-Net,” where the connectivity with respect to the original architecture is enhanced on the encoder side. In this work, the model takes as input the perfusion maps (in particular, CBV, CTP, DWT, and MTT) and outputs the segmentation of the infarcted tissue. Besides, architecture is further modified by introducing inception modules in the encoder.

Segmentation of ischemic stroke lesions from MRI imaging is the task tackled by Kadry et al. (2021). Leveraging the ISLES 2015 database, a U-Net architecture is trained to learn and extract the segmentation of the ischemic stroke lesions. Differently from the original U-Net architecture, the authors leverage over the VGG-11 backbone having on the encoder side 32, 64,128, 256, and 512 filters in the various depth stages (which are then mirrored in the decoder).

Very recently, Li et al. (2021b) have proposed a multi-scale U-Net model for ischemic stroke lesion segmentation. The authors here combine multiple convolution kernel sizes in the same layer, similarly to inception modules and as seen in the work from Dolz et al. (2018), but they also introduce the knowledge of dilated convolution which allows for extracting non-strictly local information.

In order to assess performance on segmentation of ischemic stroke lesions, Pinheiro et al. (2019) provided an extensive study on many configurations of U-Net and V-Nets, applied to relatively small datasets of MRI and CT images. The authors showed that deeper U-Nets perform better than shallow ones, and that including CT modality improves the results. Finally, they showed that employing perfusion maps yields much better results than using raw perfusion data alone.

In our work, we leverage the key messages from Pinheiro et al. (2019) to focus on the following question: can a neural network model produce perfusion maps that allow for a more accurate segmentation afterward? To answer this question, we cannot base our work on the existing literature, which has employed the U-Net model as a black box to extract the segmentation of the ischemic lesion from the raw perfusion or from the already-processed maps. Instead of using the U-Net model to solve the segmentation problem, we employ it to solve the regression problem of matching the perfusion maps. These contain the information needed by the medical expert to predict the extension of the ischemic lesion.

## 5. Conclusion

The proposed CNN-based method generated informative, AIF-independent perfusion maps of patients with AIS, approximating perfusion mismatch in brain tissues very well. Our ML model performed similarly to the state-of-the-art NLR perfusion analysis methods used as GT in estimating CBF, CBV, and TTP parametric maps.

More frequent use of ML methods for perfusion analysis can lead to the reduction of data inputs needed for perfusion mismatch prediction and therefore to a smaller radiation dose for the patients. In the near future, combining different ML approaches to CTP analysis and integrating clinical parameters in the model, has the potential to bring new improved standards in terms of acquisition protocols and ischemic core prediction.

## Data availability statement

The datasets presented in this study can be found in online repositories. The names of the repository/repositories and accession number(s) can be found below: The datasets generated for this study can be found in the Zenodo repository doi: 10.5281/zenodo.481760 and IEEE DataPort repository doi: 10.21227/x8ea-vh1.

## Ethics statement

The studies involving human participants were reviewed and approved by the Comitato Etico Interaziendale, CEI, Città della Salute e della Scienza, Turin, Italy, id number 596.345. Written informed consent for participation was not required for this study in accordance with the national legislation and the institutional requirements.

## Author contributions

UG, FD’A, ET, MG, EB, GV, and MB contributed to the conception and design of the study. UG, FD’A, ET, FB, AS, EB, AB, and RR organized the database. UG, ET, FB, AS, EB, AB, and RR performed the statistical analysis. UG, FD’A, and MB wrote the first draft of the manuscript. UG, FD’A, ET, RR, MG, EB, AB, and MB wrote sections of the manuscript. All authors contributed to the manuscript revision, read, and approved the submitted version.

## Footnotes

1
https://deephealth-project.eu/

2
https://pingouin-stats.org/build/html/generated/pingouin.intraclass_corr.html
